# Hospitalization readmission rates in patients with schizophrenia: A nationwide analysis

**DOI:** 10.1192/j.eurpsy.2023.332

**Published:** 2023-07-19

**Authors:** M. Gonçalves Pinho, J. P. Ribeiro, L. Fernandes, A. Freitas

**Affiliations:** 1 Faculty of Medicine of the University of Porto; 2 CINTESIS, Porto; 3 Centro Hospitalar do Tâmega e Sousa, Penafiel, Portugal; 4 Faculty of Medicine of the University of Porto, Porto

## Abstract

**Introduction:**

Schizophrenia is a chronic severe mental disorder characterized by acute decompensation episodes that may lead to hospitalization. In Portugal a previous study found a total of 25,385 hospitalizations in an 8-year period, being one of the most burdensome serious mental disorders in Portugal.Rehospitalizations (hospitalization occurring after a previous discharge due to Schizophrenia) are one of the quality-of-care indicators of schizophrenia treatment.

**Objectives:**

This project aims to describe and quantify hospitalization readmission rates in patients with schizophrenia in Portuguese public hospitals

**Methods:**

A descriptive study was designed according to the RECORD guidelines, using a nationwide hospitalization administrative database that contains all hospitalizations registered in Portuguese mainland public hospitals. All episodes with discharges occurring between 2008 and 2015 with a primary diagnosis of Schizophrenia were selected according to the International Classification of Diseases version 9, Clinical Modification (ICD-9-CM) codes of diagnosis 295.xx. Readmission rates were estimated using a methodological approach developed by the authors that identified patients who have been rehospitalized in <=5; <=30; <=90 and <=365 days from a previous hospitalization episode during the study period. Individual patients were identified (crosschecking three variables: birthdate; sex and place of residence). The time between discharges was calculated using the difference between an index hospitalization and the next registered hospitalization from the same patient.

**Results:**

A total of 14,279 patients were anonymously identified in order to calculate readmission rates per patient from a total of 25,385 hospitalization episodes. The mean hospitalization per patient ratio was 1.78. A total of 367 patients (2.6%) had a readmission in <=5 days after discharge. The readmission rate at <=30 days was 8.6% (n=1224); 14.1% (n=2013) at <= 90 days and 23.7% (n=3378) at <=365 days. Readmission rates were higher in male sex patients. Shorter periods of time between readmissions were increasingly frequent in male patients (3.1% vs. 1.6% of all male vs. all female patients in <=5days readmissions; 9.6% vs. 6.5% in <=30 days readmissions; 15.7% vs. 11.0% in <=90days readmissions and 25.3% vs. 20.4% in <=365days readmissions).

**Conclusions:**

Rehospitalizations arise as one of the indicators of treatment failure and quality of care in patients with a diagnosis of schizophrenia. Our study is the first to measure and assess readmission rates in patients with Schizophrenia in Portuguese public hospitals at a nationwide level. Portugal presents lower 30-day readmission values when compared to other countries. The 30-day readmission rate in patients with Schizophrenia in Portuguese Public Hospitals is 8.6% and male patients have higher readmission rates when compared to female patients.

**Disclosure of Interest:**

None Declared

